# Ipecacuanha

**Published:** 1888-03

**Authors:** L. B. Bouchelle

**Affiliations:** Thomasville, Ga.


### IPECACUANHA.

By L. B. BOUCHELLE, M. D., THOMASVILLE, GA.

Since 1648 this drug has been in the armamentarium of the profession, and has had its days of popularity, and, unfortunately, of decline. It was, for a time, held as a secret remedy in France, and used with great success by a French physician, so much so, that one of the Louis family gave a large sum of money to John Helistius to reveal its name.

Its "taking" properties were exerted, at that early date, in dysentery and other diseases of the bowels. Its general properties are too well known to the profession, to require even an enumeration. This writing is more for the purpose of inviting the attention of medical men to its virtues, even now, in a great variety of ailments, and have it, as it were, brought to the front, than anything else. It has as wide a field of application, likely, as any other article in the materia medica. It is indicated in all the diseases affecting the lungs, bronchi, and fauces. It reaches hepatic complications most happily. There is nothing superior to it in alvine troubles. And when we come to the great manufacturing organ of the system, the stomach, it is par excellence, the drug, the *remedium principale.* But to particularize: We are called to a case of pneumonia in the first stage. It is *the* remedy to begin with—a brisk emetic. Then to promote and facilitate expectoration, combine it with *every dose* given while treating the case, with due discrimination, as to the quantity. With tartarized antimony, and quinine, and, of course, it is in the soporific at night, in Dover powders. In bronchitis, it is equally indicated in all its stages and types as diaphoretic, revulsive, alterative and expectorant. In croup, it is the remedy to begin, continue and end with. The writer prefers the syrup in croup. And then there is diphtheria, the fell destroyer of its many victims, that the writer would feel at a loss to treat successfully without it.

Suffer a paragraph by way of parenthesis, to let me say, that if the practitioner will vomit his cases of diphtheria freely with ipecac, and apply pure, undiluted carbolic acid to he ulcers, and then keep the sufferer in sight of nausea with small doses of ipecac, chlorate of potash and carbolic acid, he will feel proud, and the patient's friends will praise him for his skill. Albeit, *nothing* will relieve the sufferer if it has been let alone too long.

The following prescription is my favorite:

| | | |
|---|---|---|
| ℞. | Water | O. j |
| | Ipecac | grs xx, |
| | Quinine | grs. x, |
| | Carbol c acid | gtts. xx. |

M.

Of this give tablespoonful every twenty minutes, till emesis is fully produced, then empty the bowels with calomel or blue mass. After this teaspoonful doses one or two hours apart most of the day; gargle with the following at will:

| | | |
|---|---|---|
| ℞. | Chlorate potash | grs. c, |
| | Carbolic acid | gtts. xxx, |
| | Water | O. ss. |

At night give Dover powders; dose to suit the age.

With this treatment, *all cases*, seen in time, recover in my hands. In icterus (jaundice), ipecac is a sheet anchor. Look at him—orange-tinged skin, yellow-eyed, shrunken features, with no energy, perspitory glands all sealed, alvine discharges chalky; wants nothing to eat, nothing to drink, with an air that indicates that money and friends have taken the last off-bound train, never to return. What M. D. has not had this spectre haunt him as he trudges the weary way homeward? Well, fellow, heart-sick and weary, pour into him a pint of warm water, with 20 to 30 grains of ipecac suspended, and after he is well washed out with repeated draughts of water as hot as can be borne, let the stomach rest two or three hours, and give an adult 10 to 20 grains blue mass, and your patient will eat the first meal after its action, the liver will go to work, and "all is well."

And these fevers: so-called malarial, remittent, topho-malarial—a misnomer—and typhoid, *when seen in time*, premise all other drugs, with a brisk emetic of ipecac.

Is this O. S.? Nevertheless it is the *surest* way to relieve and the shortest road to convalescence. Put small quantities of ipecac in each dose of quinine, enough to determine to the skin, and provoke the liver to healthy secretion.

Here is a case, the like of which we all frequently encounter: "Dr. I have been sick, up and down, for six or eight weeks, sometimes up a few days, then down, and I have taken quinine and compound cathartic pills repeatedly, and can out eat a railspliter, and am no count." Puke him well, give no more c. c. pills (*execrable things*), but some mercurial cathartics, then, in each dose of quinine, enough ipecac to *destroy* that morbid appetite, and his remedy is an accomplished fact.

Then these miserable cases of recurrent intermittents that bring us all into disrepute so often, what shall we do to break them up? The writer has used the following prescription, in this class of cases, for nearly twenty years, varying the quantities of the drugs, according to age, and *sex*, and *race*, with unqualified success:

℞. Quinine.................................ʒ iv,

Iron by hydrogen..........................ʒ iv,

Strychnia ...................................grs. iv,

Arsenic......................................grs. iv,

Ipecac.......................................grs. iv.

M. And make into 60 pills. Take one after each meal. Women require lesss than men; negroes more than white folks.

What a tide of dyspeptics there is in all the country! And what a set of gormandines they all are! Empty their stomachs *well* with ipecac, rest them a few hours, and follow each meal with an acid or an alkali, and just enough ipecac not to vomit, and Mr. Gorman will become more dainty and delicate, eat less, grunt less and sleep more, and keep his irritable temper in sweet subordination to a becalmed judgment.

Too much to eat, too little to do, and too much temporizing and extemporizing are the great curses of the day. Give them all enough ipecac to bring physical and metaphysical forces on an equilibrium.

